# Analytic attributes of the 16S rRNA gene sequencing methodology for human gut microbiota characterization

**DOI:** 10.1007/s00253-026-13844-8

**Published:** 2026-05-22

**Authors:** Diego A. Rivera, Oscar J. Lara-Guzmán, N. Andrea Villota-Salazar, Jelver A. Sierra, Katalina Muñoz-Durango, Juan S. Escobar

**Affiliations:** 1 Vidarium–Nutrition, Health, and Wellness Research Center, Grupo Empresarial Nutresa, Carrera 52 #2-38, Medellin, 050024 Colombia; 2 Present Address: Faculty of Medicine, Universidad de Antioquia, Carrera 51D #62- 29, Medellin, 050010 Colombia

**Keywords:** Microbiome, Precision, Repeatability, Intermediate precision, Limit of quantification, Standardized operating procedure

## Abstract

**Abstract:**

Gut microbiota is crucial for human health. While 16S rRNA gene sequencing is most used for characterizing this community, the validation and standardization of the technique are often overlooked. This study analyzes critical factors influencing the repeatability and intermediate precision of 16S rRNA gene sequencing methodology for human gut microbiota characterization, examining the impact of key analytical factors. Our investigation evaluated the effects of the DNA extraction protocol, sample homogenization, thawing, library preparation, and sequencing on measurements of precision. This established a standardized operating procedure (SOP) whose variability was assessed within a single laboratory (intermediate precision) by analyzing DNA extraction kit lot variations and the laboratory analyst handling the samples. We discovered that the DNA extraction protocol and sample thawing were the most significant drivers of variability in gut microbiota profiles. At the same time, the intermediate precision of the method was high. We determined the method’s limit of quantification, revealing an impressive sensitivity down to just 11 to 18 rarefied read counts (with coefficients of variation of 30% and 20%, respectively). Beyond technical considerations, we also quantified the variation in gut microbiota profiles among individuals and over time. Our findings confirm substantial inter-individual differences while demonstrating that changes within individuals over a week are relatively small. This research illuminates some critical factors influencing the precision and consistency of 16S rRNA gene sequencing for gut microbiota analysis. By incorporating these insights into standardized protocols, we can significantly improve best practices in DNA sequencing methodologies, strengthening the reliability and comparability of human microbiome studies.

**Key points:**

• *DNA extraction and sample thawing critically affect the method’s precision.*

• *We established an SOP with high repeatability, intermediate precision, and a specific limit of quantification.*

• *Gut microbiota profiles substantially vary among individuals but remain stable over a week.*

**Supplementary Information:**

The online version contains supplementary material available at 10.1007/s00253-026-13844-8.

## Introduction

The human gut microbiota, comprising trillions of microbial cells of hundreds of species, plays a pivotal role in human health and disease (Fan and Pedersen 2020). Characterizing these microbial communities has been revolutionized by high-throughput sequencing technologies, particularly the 16S rRNA gene sequencing methodology (Tringe and Hugenholtz 2008; Caporaso et al. 2011). This approach targets fast-evolving, hypervariable regions of the 16S rRNA gene, allowing for the assessment of diversity and taxonomic profiling of complex microbial communities without needing cultivation (Parks et al. 2022). The popularity of 16S rRNA gene sequencing in microbiome research stems from its cost-effectiveness, scalability, and ability to provide insights into microbial diversity and composition (Caporaso et al. 2011) and even potential function (Douglas et al. 2020).

Despite its widespread use, the 16S rRNA gene sequencing methodology faces challenges affecting data precision and reproducibility. While efforts have been made to standardize bioinformatics pipelines for reproducibility data analysis and interpretation (Kozich et al. 2013; Bolyen et al. 2019; Straub et al. 2020), other fronts have received less attention. This includes factors affecting repeatability, that is, the consistency of measurements when the same sample is analyzed multiple times, such as sample storage (Cardona et al. 2012), DNA extraction (Elie et al. 2023), sample processing (Santiago et al. 2014), and library preparation and sequencing (Tourlousse et al. 2021). All these factors can introduce variability in results, impacting the reliability of microbiome studies and complicating comparisons across different studies and laboratories (Tourlousse et al. 2021). Importantly, these factors affect not only 16S rRNA gene sequencing but all methodologies for microbiome study (*e.g.*, metagenomics, meta-transcriptomics).

Unfortunately, validation and standardization in microbiome research has not been common. Most papers lack information about validation of molecular-biology protocols, the inclusion or not of positive and negative controls, the way of accounting and removing potential contaminants, reporting on sequencing error rates, limits of quantification, among others. There is a growing need to establish standardized protocols and quality control measures to address the mentioned challenges and enhance the robustness of 16S rRNA gene sequencing for human gut microbiota characterization (Tourlousse et al. 2017, 2018, 2021, 2022; Vera-Wolf et al. 2018; Elie et al. 2023). Such standards would aim to minimize and quantify technical variability, improve methodological transparency, and ensure the reproducibility of findings across laboratories. By evaluating several key analytic attributes of the 16S rRNA gene sequencing methodology for human gut microbiota characterization in the same study, we seek to quantify the method’s variability and its limit of quantification (LOQ), thus contributing to developing standards that optimize its precision and, ultimately, reliability in microbiome research.

## Materials and methods

### Samples

This study analyzed fecal samples from four self-declared healthy individual donors (one female and three males) obtained in October 2022. Three participants donated 7 samples and one donated 2 samples, obtained over a week. Participants refrigerated the collected feces in portable freezers with gel packs provided by the research team and sent them to our laboratory within 4 h. All participants signed a written informed consent.

### Experimental settings

**Fig. 1:**
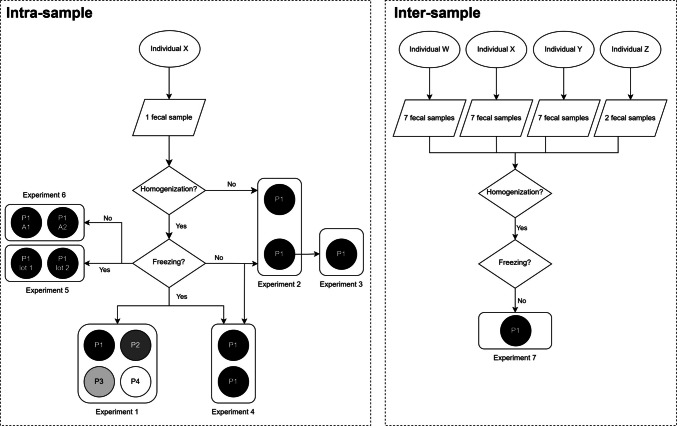
Flowchart of the different experiments to quantify intra-sample and inter-sample variability of the 16S rRNA gene sequencing methodology for human gut microbiota characterization. P1: protocol 1 (Qiagen DNeasy Blood & Tissue kit, adapted protocol for animal fecal tissues); P2: protocol 2 (Qiagen DNeasy Blood & Tissue kit, protocol to maximize DNA from Gram-positive bacteria); P3: protocol 3 (Qiagen QIAamp PowerFecal Pro DNA kit, following the manufacturer’s instructions); P4: protocol 4 (ZymoBIOMICS DNA Microprep kit, following the manufacturer’s instructions); *A1* laboratory analyst 1, *A2* laboratory analyst 2

We performed several experiments to quantify intra-sample and inter-sample variability in gut microbiota alpha diversity, beta diversity, and microbial abundance from different sources (Fig. 1).

*Experiment 1* quantified the variability introduced by the DNA extraction protocol. A participant’s fresh fecal sample was thoroughly homogenized with an electric stirrer, aliquoted, and frozen at −80 °C. DNA from thawed aliquots (replicates) was extracted using four DNA extraction protocols and three commercial kits: (i) protocol 1: DNeasy Blood & Tissue Kit (Qiagen, Hilden, Germany) adapted for animal fecal tissues (Shanmuganandam 2019) (*n* = 3 replicates); (ii) protocol 2: DNeasy Blood & Tissue Kit, following the manufacturer’s protocol to maximize DNA from Gram-positive bacteria (*n* = 3); (iii) protocol 3: QIAamp PowerFecal Pro DNA Kit (Qiagen, Hilden, Germany), following the manufacturer’s instructions (*n* = 3); (iv) protocol 4: ZymoBIOMICS DNA Microprep Kit (Irvine, CA, USA), following the manufacturer’s instructions (*n* = 3). These four protocols were chosen because of their ample use in the literature. The DNeasy Blood & Tissue Kit is a classic kit for general molecular biology, that has been optimized for the study of the human gut microbiome, including a dedicated bead-beating step to avoid overrepresentation of easy-to-lyse Gram-negative bacteria (Shanmuganandam 2019). The QIAamp PowerFecal Pro DNA Kit is commonly used in fecal metagenomics (Lim et al. 2020) and has been reported to recover a significantly higher proportion of Gram-positive bacteria compared to older chemical-only methods (Lim et al. 2018). The ZymoBIOMICS DNA Microprep Kit has been validated against a mock community (Rauer et al. 2025) and is regarded as one of the most accurate kits for maintaining the “true” relative abundance of a sample. A single analyst performed DNA extractions in the same laboratory on the same day, using a single lot of DNA extraction kit per extraction.

*Experiment 2* assessed the effect of sample homogenization. A participant’s fresh fecal sample was aliquoted at different points (*n* = 5, non-homogenized replicates). The remaining feces were thoroughly homogenized and aliquoted (*n* = 6, homogenized replicates). DNA of homogenized and non-homogenized replicates was extracted using protocol 1. A single analyst performed DNA extractions in the same laboratory, on the same day, using a single lot of the DNA extraction kit.

*Experiment 3* calculated the variability introduced at library preparation and DNA sequencing. Instead of analyzing different library preparation methods and sequencing technologies, this experiment aimed to quantify technical variability within an established and publicly available SOP. Thus, library preparation and sequencing were analyzed as a single factor. DNA extracted from a single homogenized aliquot of *Experiment 2* was split (*n* = 5 replicates) and used as the template for PCR amplification, library preparation, and sequencing.

*Experiment 4* measured the effect of sample thawing. In this experiment, a homogenized fecal sample of one individual was aliquoted. DNA was extracted from fresh (*n* = 6 replicates) or thawed aliquots maintained at −80 °C for a week (*n* = 6). DNA was extracted using protocol 1. A single analyst performed DNA extractions in the same laboratory, on the same day, using a single lot of the DNA extraction kit.

*Experiment 5* quantified the variability introduced by the DNA extraction kit lot. For this, homogenized aliquots of a single fecal sample of one individual were kept at −80 °C for a week. DNA was extracted from thawed aliquots using two different lots of the DNeasy Blood & Tissue Kit employed in protocol 1: lot 1 (ref. 169043862; *n* = 6 replicates) and lot 2 (ref. 169050541; *n* = 6). A single analyst performed DNA extractions in the same laboratory on the same day.

*Experiment 6* measured the variability introduced by the laboratory analyst. For this, a single homogenized fresh fecal sample was aliquoted and processed by two analysts: A1 (*n* = 6 replicates) and A2 (*n* = 6). DNA was extracted using protocol 1. Extractions were performed in parallel by the two analysts, in the same laboratory, on the same day.

*Experiment 7* quantified inter-sample variability, that is, among individuals and time (defecations). Fresh samples of the four individual donors were homogenized and aliquoted. A single analyst in the same laboratory extracted DNA from two replicates per individual and time with protocol 1.

Total DNA from the seven experiments was quantified using fluorometry and stored at −20 °C. Samples were diluted to 3 ng/µL, randomized, and sent to the University of Michigan’s Microbiome Core (Ann Arbor, MI, USA) for PCR amplification, library preparation, and sequencing. The SOP for library preparation and sequencing was previously described (Seekatz et al. 2015) and is publicly available (https://github.com/SchlossLab/MiSeq_WetLab_SOP). Briefly, the V4 region of the 16S rRNA gene was amplified using a set of barcoded dual-index primers (F515 and R806), as previously described (Kozich et al. 2013). Each 20-μL PCR was composed of 5 µL of a 4 µM equimolar primer set, 0.15 µL of Accuprime High-Fidelity Taq polymerase, 2 µL of 10 × Accuprime PCR buffer II (Life Technologies, Carlsbad, CA, USA; catalog no. 12346094), 11.85 µL of sterile PCR-grade water, and 1 µL of DNA template. The PCR cycle consisted of 2 min at 95 °C, followed by 30 cycles of 95 °C for 20 s, 55 °C for 15 s, and 72 °C for 5 min, followed by 72 °C for 10 min. PCR products were visualized using an E-Gel 96 with SYBR Safe DNA Gel Stain 2% (Life Technologies, Carlsbad, CA, USA; catalog no. G7208-02). A SequelPrep normalization plate kit (Life Technologies, Carlsbad, CA, USA; catalog no. A10510-01) was used to normalize the DNA library to the lowest concentration of the pooled plates. The Kapa Biosystems Library Quantification kit for Illumina platforms (Kapa Biosystems, Cape Town, South Africa; catalog no. KK4824) was used to determine the pooled library concentration, and the Agilent Bioanalyzer high-sensitivity DNA analysis kit (Agilent Technologies, Santa Clara, CA, USA; catalog no. 5067-4626) was used to determine the amplicon size. Amplicons were sequenced on the Illumina MiSeq platform using a MiSeq Reagent kit v2 (catalog no. MS-102–2003) for 500 cycles according to the manufacturer’s instructions with modifications for the primer set. Libraries were prepared according to Illumina’s protocol “Preparing Libraries for Sequencing on the MiSeq” (part 15039740, Rev. D) for 2 nM libraries. The final load concentration was 5.5 pM, spiked with 15% PhiX to add diversity. Sequencing reagents were prepared according to the “16S Sequencing with the Illumina MiSeq Personal Sequencer” protocol with custom read 1/read 2 and index primers added to the reagent cartridge, generating FASTQ files with paired-end reads. As a positive control, we added a mock community (ZymoBIOMICS Microbial Community DNA Standard, Zymo Research). We included two types of negative controls: (i) extraction blanks to control for potential contamination during DNA isolation; this is because it has been shown that DNA extraction kits are not DNA-free and can severely affect the microbiome inference (Salter et al. 2014); (ii) ultrapure water, to control for contamination introduced during PCR and sequencing.

### Bioinformatics and statistical analyses

Raw reads were processed with Mothur v.1.46.1 using the Illumina MiSeq standard operating procedure (Kozich et al. 2013). We extracted sequences and quality scores from paired FASTQ files and assembled the reads to form contigs. We eliminated sequences containing non-ACGT bases and sequences shorter than 275 bp. Next, we aligned the sequences using the Silva v123 reference alignment (Quast et al. 2013). We removed sequences with a homopolymer run greater than or equal to 8 nucleotides and sequences that did not overlap with the alignment region spanning the V4 hypervariable region of *Escherichia coli* (GenBank: J01859.1). Then, we performed a pre-clustering step in which sequences differing in one or two nucleotides were merged. We detected and discarded chimeric sequences with UCHIME (Edgar et al. 2011). After that, we assigned taxonomic classifications to the sequences using Silva v123 and removed sequences classified as chloroplast, mitochondria, eukaryote, or unknown. We generated operational taxonomic units (OTUs) delimited at 97% identity with the OptiClust algorithm (Westcott and Schloss 2017). OTUs were taxonomically classified as per GTDB v220 (Parks et al. 2022), using SATIVA with vetted 16S rRNA sequences (Lundin and Andersson 2022). Potential contaminating sequences detected from negative controls were removed with Decontam (Davis et al. 2018). The OTU table, taxonomy, and metadata served to create phyloseq objects. The OTU table was rarefied, and read counts were transformed using centered log-ratios (CLR). Before CLR transformation, we applied a pseudo-count of min(relative abundance)/2 to zero counts using the microbiome R package’s *transform* function (Lahti 2012) . Alpha diversity was evaluated through richness, Pielou’s evenness, and the Shannon diversity index. Beta diversity was assessed by Aitchison distances (*i.e.*, Euclidean distances of CLR-transformed data). Alpha and beta diversities were evaluated at the phylum, class, order, family, genus, species, and OTU levels.

Statistical analyses were performed in R 4.5.1 (R Core Team 2019). For alpha diversity, the Shapiro–Wilk and Fligner-Killeen tests were performed to test normality and homogeneity of variance, respectively. Welch’s *t*-test or ANOVA compared alpha-diversity indices per categorical sources of variability, as appropriate. Aitchison distances were calculated and visualized through principal components analysis (PCA). Permutational analyses of variance (PERMANOVA) with 1000 permutations measured the effect of each experiment’s source of variability on the total variance. Differences in microbial abundance were evaluated with ALDEx2 (Fernandes et al. 2013). CLR abundances were compared between categorical sources of variability at all taxonomic levels through Welch’s *t*-test. *P*-values were adjusted for multiple comparisons using the Benjamini–Hochberg correction. In all cases, we calculated coefficients of variation ($CV=\frac{\sigma }{\mu }$, where *σ* is the standard deviation and *µ* is the mean) among appropriate replicates. Spearman correlation coefficients were obtained between CLR abundance and CV, where appropriate.

We calculated the method’s LOQ using DNA extracted from different homogenized aliquots from *Experiment 2*. We grouped sequences at the OTU level, rarefied the dataset to 20,000 reads/sample, and calculated CLR abundances. OTUs with negative CLR were removed (*i.e.*, OTUs with very low read counts). Next, we fitted the curve and obtained the equation between (log) CV and OTU abundance (log-rarefied read counts or CLR). According to the fitted curve, the LOQ was defined as the OTU abundance at 30%, 20%, or 10% CV.

## Results

We evaluated some key analytic attributes of the 16S rRNA gene sequencing methodology for human gut microbiota characterization to establish standards that improve its precision and reproducibility. We assessed critical factors affecting the method’s repeatability and intermediate precision and calculated its LOQ. Next, we quantified microbiome profiles among individuals and over time (Fig. 1).

### DNA extraction protocol

**Fig. 2:**
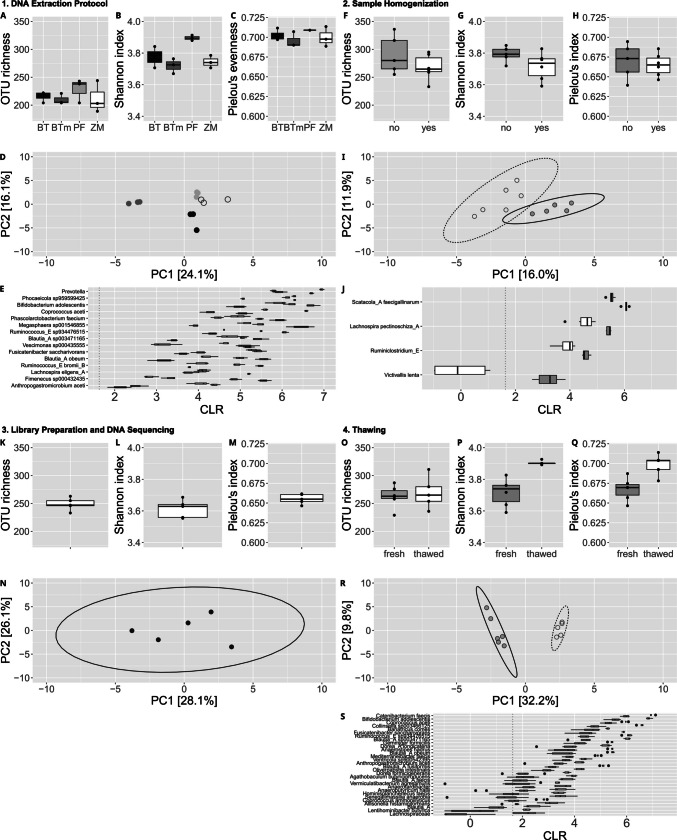
Repeatability of the 16S rRNA gene sequencing methodology for human gut microbiota characterization. Variability due to (1) the DNA extraction protocol, (2) sample homogenization, (3) library preparation and DNA sequencing, and (4) sample thawing. Alpha diversity was estimated through OTU richness (panels **A**, **F**, **K**, **O**), Shannon diversity index (panels **B**, **G**, **L**, **P**), and Pielou’s evenness index (panels **C**, **H**, **M**, **Q**). For each evaluated condition, the box-and-whisker plot depicts the lower quartile, the median, and the upper quartile, and the points indicate each replicate. Beta-diversity principal component analysis based on Aitchison distances at the OTU level (panels **D**, **I**, **N**, **R**). The percentages on the axes represent the proportion of explained variance. Ellipses encompass 95% of the replicates for each condition. Point colors represent the same condition indicated in the upper panels. OTUs with significantly different abundances (panels **E**, **J**, **S**). The box-and-whisker plot depicts the lower quartile, the median, and the upper quartile. Box colors represent the same condition indicated in the upper panels. Dotted vertical line: limit of quantification for a CV of 30% (see Fig. 4B). BT: protocol 1 (Qiagen DNeasy Blood & Tissue kit, with an adapted protocol for animal fecal tissues); BTm: protocol 2 (Qiagen DNeasy Blood & Tissue kit, with a protocol to maximize DNA from Gram-positive bacteria); PF: protocol 3 (Qiagen QIAamp PowerFecal Pro DNA kit, following the manufacturer’s instructions); ZM: protocol 4 (ZymoBIOMICS DNA Microprep kit, following the manufacturer’s instructions). *CLR* centered log-ratio

We compared four protocols for DNA extraction using three commercial kits of ample use. The alpha-diversity analyses indicated no significant differences in richness at any taxonomic level among the protocols (Fig. 2A). In contrast, differences in the Shannon diversity index and Pielou’s evenness were significant at almost all taxonomic levels (Fig. 2B, C). Still, CV% among replicates within a single protocol was low (Table S1). The analysis of beta diversity based on Aitchison distances indicated that the DNA extraction protocol significantly impacted the gut microbial structure at all taxonomic levels and explained between 48 and 62% of the total variance (Fig. 2D; Table S1). Importantly, depending on the taxonomic level, the CV% of Aitchison distances among replicates treated with the same DNA extraction protocol was low to moderate (Table S1). In terms of abundance, we found 1 phylum, 2 classes, 4 orders, 5 families, 12 genera, 14 species, and 15 OTUs with statistically significant varying abundances among protocols. This mainly were Gram-positive bacteria from phyla *Bacillota_A*, *Bacillota_C*, and *Actinomycetota*, including genera *Anthropogastromicrobium*, *Blautia_A*, *Coprococcus*, *Fimenecus*, *Fusicatenibacter*, *Lachnospira*, *Ruminococcus_E*, *Vescimonas*, *Megasphaera*, *Phascolarctobacterium*, and *Bifidobacterium* (Fig. 2E; Supplementary File 2).

### Sample homogenization

Feces homogenization did not produce significant differences in alpha diversity at any taxonomic level. CV for alpha-diversity estimates was low among homogenized and non-homogenized replicates: <12% for richness and <5% for Shannon and Pielou indices (Fig. 2F–H; Table S2). On the other hand, PERMANOVA on Aitchison distances indicated that sample homogenization significantly impacted beta diversity at all taxonomic levels. The lower the rank of the taxonomic gathering, the smaller the impact of sample homogenization on the total variance. Sample homogenization explained up to 40% of the gut microbial structure at the class level and down to 15% at the OTU level (Fig. 2I). Despite this, Aitchison distances were generally similar among homogenized and non-homogenized replicates, and CV was <10% at the family, genus, species, and OTU levels (Table S2). Sample homogenization also affected microbial abundance, although in a limited number of taxonomic groups. One phylum, class, and order, 4 families, 7 genera, 5 species, and 4 OTUs had significantly different abundances between homogenized and non-homogenized replicates. These corresponded to Gram-negative bacteria from phyla *Verrucomicrobiota* and *Pseudomonadota*, and Gram-positive *Bacillota_A* (Fig. 2J; Supplementary File 3).

### Library preparation and DNA sequencing

The analysis of the five replicates of the same DNA extracted from a single homogenized aliquot that underwent library preparation and sequencing following an established SOP indicated that alpha-diversity indices had CV < 10% at all taxonomic levels (Fig. 2K–M; Table S3). Beta diversity had CV < 5% at the genus, species, and OTU levels but was higher at the family, order, class, and phylum levels (Fig. 2N; Table S3). The ALDEx2 analysis indicated that microbial abundance was inversely correlated with CV (all Spearman correlation tests had *p* < 0.05; Supplementary File 4).

### Thawing

We removed one thawed aliquot that produced anomalous data for unknown reasons. The analysis was performed on six fresh and five thawed replicates. It indicated that thawing significantly affected alpha diversity at several taxonomic levels. It increased microbial evenness, although the magnitude of the differences was small. CV among fresh and thawed replicates was <5% for Shannon and Pielou indices, and <15% for richness (Fig. 2O–Q; Table S4). PERMANOVA on Aitchison distances indicated that thawing significantly affected beta diversity at all taxonomic levels. The explained variance due to the sample’s temperature cycling diminished at lower taxonomic levels. It was as high as 73% at the class level and as low as 32% at the OTU level. CV among fresh and thawed replicates diminished similarly, with relatively low (<15%) and comparable values at the family, genus, species, and OTU levels (Fig. 2R; Table S4). The ALDEx2 analysis indicated that thawing extensively impacted microbial abundance. Seven phyla, nine classes, 15 orders, 19 families, 56 genera, 56 species, and 31 OTUs had significantly different abundances between fresh and thawed replicates. This mainly included Gram-positive bacteria from phyla *Bacillota_A* and *Actinomycetota* (Fig. 2S; Supplementary File 5).

### DNA extraction kit lot

**Fig. 3:**
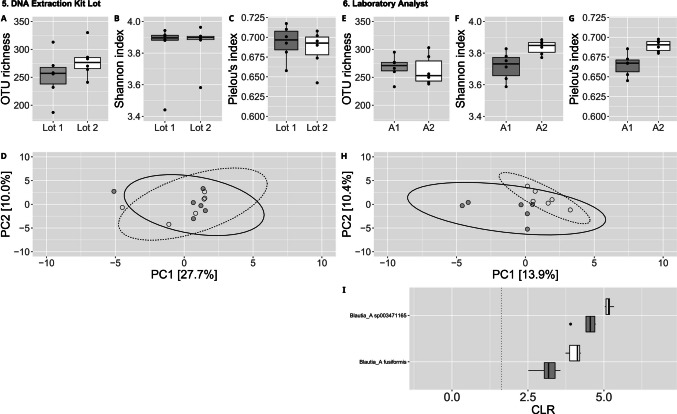
Intermediate precision of the 16S rRNA gene sequencing methodology for human gut microbiota characterization. Intra-laboratory variability due to (5) the DNA extraction kit lot and (6) the laboratory analyst. Alpha diversity estimated through OTU richness (panels **A**, **E**), Shannon diversity index (panels **B**, **F**), and Pielou’s evenness index (panels **C**, **G**). For each evaluated condition, the box-and-whisker plot depicts the lower quartile, the median, and the upper quartile, and the points indicate each replicate. Beta-diversity principal component analysis based on Aitchison distances at the OTU level (panels **D**, **H**). The percentages on the axes represent the proportion of explained variance. Ellipses encompass 95% of the replicates for each condition. Point colors represent the same condition indicated in the upper panels. OTUs with significantly different abundances (panel **I**). The box-and-whisker plot depicts the lower quartile, the median, and the upper quartile. Box colors represent the same condition indicated in the upper panel. Dotted vertical line: limit of quantification for a CV of 30% (see Fig. 4B). *A1* analyst 1, *A2* analyst 2, *CLR* centered log-ratio

We evaluated the methodology’s intermediate precision, particularly the DNA extraction kit lot and the laboratory analyst who treated the samples. Concerning the kit lot, there were no significant differences between the two lots for the evaluated alpha-diversity indices, except for richness at the order and family levels. CV was <10% for most indices and taxonomic levels (Fig. 3A–C; Table S5). Likewise, there were no differences in Aitchison distances between the two DNA extraction kit lots. CV was <20% at the OTU level (Fig. 3D; Table S5). Also, no differences in microbial abundance were observed between the two DNA extraction kit lots at any taxonomic level (Supplementary File 6).

### Laboratory analyst

Alpha diversity between the two analysts who treated the samples differed significantly according to the Shannon and Pielou indices but not for richness. Importantly, alpha-diversity estimates and taxonomic levels were similar, and CV was <10% among replicates treated by the two analysts (Fig. 3E–G; Table S6). PERMANOVA indicated that beta diversity was significantly different between the two analysts at the genus, species, and OTU levels but not at higher taxonomic ranks. Despite this, CV was <10% at the order, family, genus, species, and OTU levels (Fig. 3H; Table S6). The differential abundance analysis indicated that the laboratory analysts’ impact was limited to one genus, two species, and two OTUs (Fig. 3I). All other taxa had similar abundances (Supplementary File 7).

### Limit of quantification (LOQ)

**Fig. 4:**
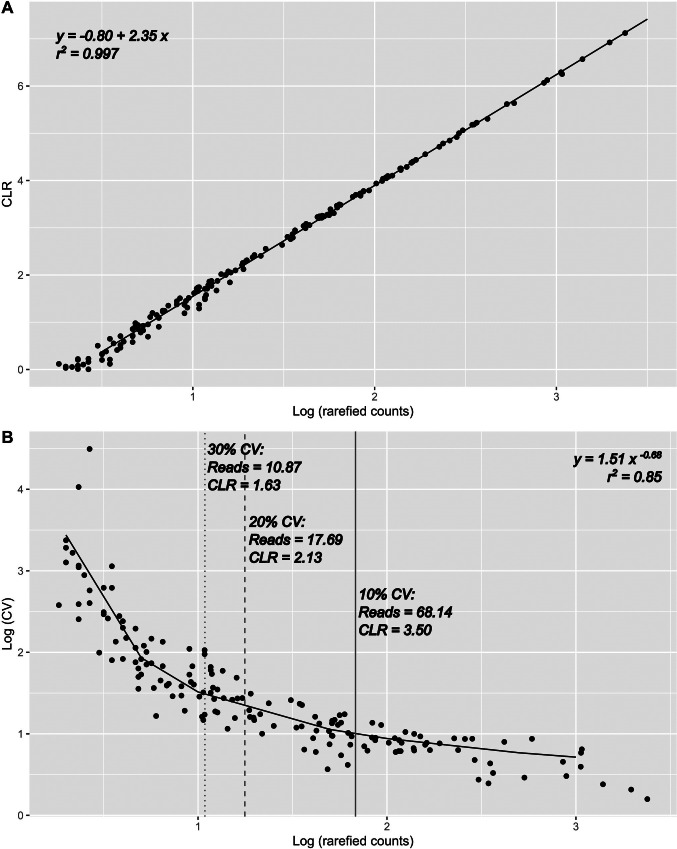
Limit of quantification (LOQ) of the 16S rRNA gene sequencing methodology for human gut microbiota characterization. **A** Linear regression between the log-rarefied OTU abundance as a function of centered log-ratio (CLR) abundance. The equation and coefficient of determination are shown. **B** Non-linear least squares fit between the log-rarefied OTU abundance and log-CV. The LOQ, that is, the OTU abundance, either as the number of rarefied read counts or CLR, is shown for three different CVs (30%, 20%, and 10%)

We determined the method’s LOQ by fitting a non-linear curve between OTU abundance and CV among replicates. Note that the results were similar if we fitted the curve with CLR or rarefied read counts, as these two measures of abundance are collinear (Fig. 4A). For a CV of 30%, the OTU abundance was 1.63 CLR or 10.87 rarefied read counts. For a CV of 20%, the OTU abundance was 2.13 CLR or 17.69 rarefied read counts. For a CV of 10%, the OTU abundance was 3.50 CLR or 68.14 rarefied read counts (Fig. 4B).

### Variability among individuals and over time

**Fig. 5:**
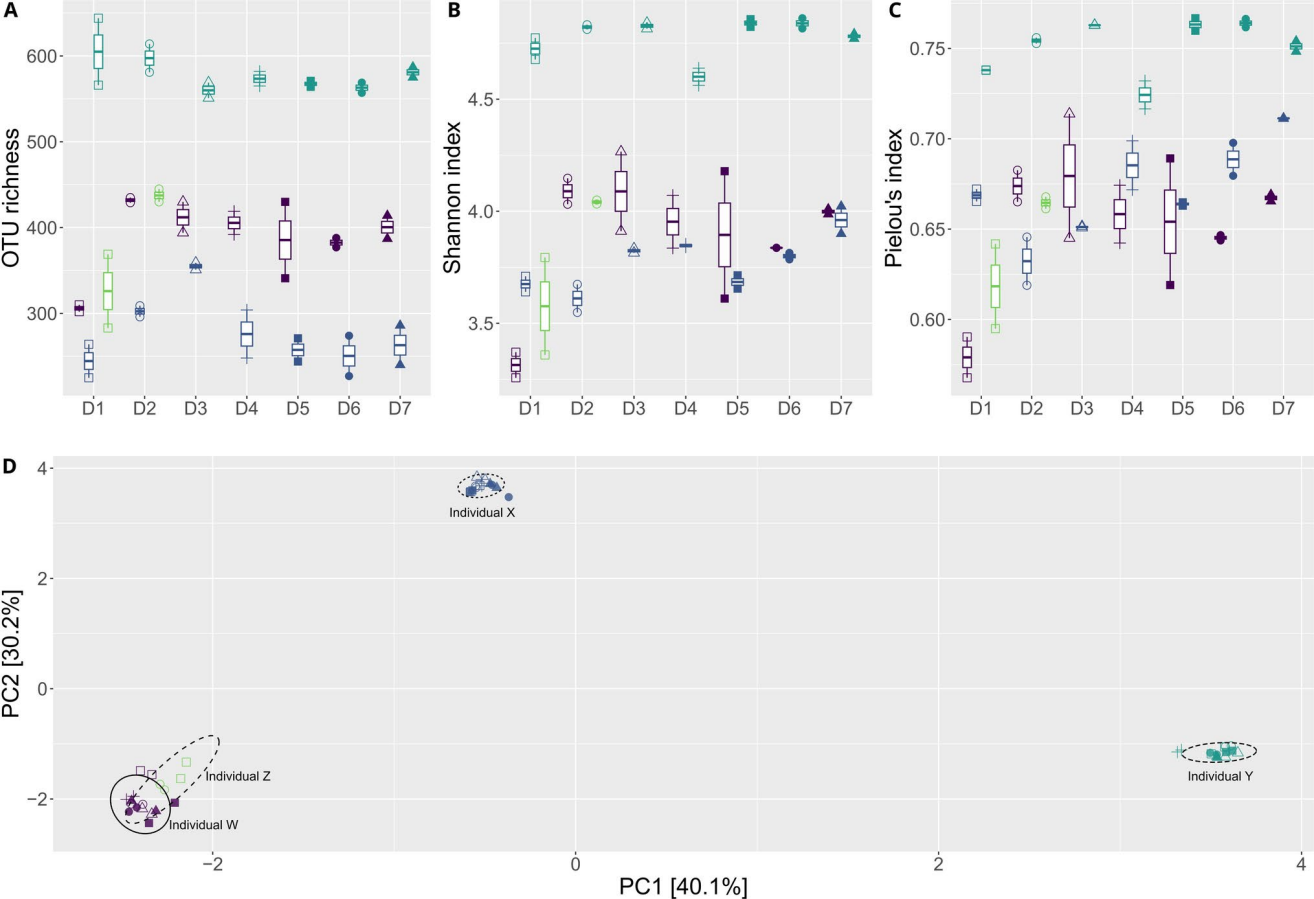
Variability among individuals and over time in gut microbiota profiles. Alpha diversity estimated through OTU richness (**A**), Shannon diversity index (**B**), and Pielou’s evenness index (**C**). The box-and-whisker plot depicts the lower quartile, the median, and the upper quartile, and the points indicate each replicate. **D** Beta-diversity principal component analysis based on Aitchison distances at the OTU level. The percentages on the axes represent the proportion of explained variance. Ellipses encompass 95% of the replicates for each individual. In all panels, samples from the same individual are depicted with similar colors, and samples from different days (D1–D7) have different shapes

Our analyses indicated that alpha-diversity indices significantly differed among individuals and over time (defecations) for most taxonomic levels (Fig. 5A–C). Of note, each individual’s time variation was low and indistinguishable from the method’s intra-sample variation described above for all indices, taxonomic levels, and subjects (Table S7). Beta-diversity variation among individuals was high and significantly explained by individual-level differences in microbial community structure. Beta diversity also varied in time for each individual, although in a smaller proportion (Fig. 5D). Within-individual Aitchison distances’ CV was highest at the phylum level and lowest at the OTU level (Table S8). The ALDEx2 analysis indicated that microbial abundance was inversely correlated with variability over time (CV among defecations) (Supplementary File 8).

## Discussion

This investigation evaluated critical factors affecting variability of the 16S rRNA gene sequencing methodology for human gut microbiota characterization. Unlike previous efforts made by the Microbiome Quality Control project, that primarily assessed variability across different laboratories and bioinformatic pipelines (Sinha et al. 2017; Costea et al. 2017), our research focused on the formal analytical validation of a specific 16S rRNA gene sequencing methodology within a single, controlled laboratory environment to ensure the method’s reliability for routine use. We focused on the method’s precision, that is, the closeness of agreement between independent test results. We demonstrated that, under our experimental conditions, the primary contributors to the method’s overall precision were the DNA extraction protocol used to obtain the template DNA and the freeze–thaw cycles applied to samples prior to DNA extraction. Other factors introduced only minor variability, including sample homogenization, library preparation and sequencing, DNA extraction kit lot, and laboratory operator.

DNA extraction protocols often vary within a laboratory to maximize yield and quality, and because commercial kits can be discontinued. The choice of the DNA extraction method is critical in microbiome studies as it affects the accuracy, precision, reproducibility, and representation of microbial communities. Indeed, DNA extraction methods can introduce variability comparable to biological differences, mimicking microbiome signatures related to health and nutrition (Bartolomaeus et al. 2021). Methods such as phenol–chloroform yield higher DNA quality and quantity, although commercial kits are more practical for high-throughput (Roager et al. 2023). It has been shown that bead-beating or enzymatic treatments can enhance DNA extraction from Gram-positive bacteria (Knudsen et al. 2016; Tourlousse et al. 2021). A systematic evaluation of 21 DNA extraction protocols with several commercial kits using a cell mock community showed excellent repeatability among replicates and comparable yields of high-molecular-weight DNA. Still, substantial variation was observed for the abundance of Gram-positive and Gram-negative bacteria (Tourlousse et al. 2021). In addition, the matrix effects were not evaluated in the mentioned study, which can introduce additional variation. These differences in microbiome inference arise from the principles of the genetic material extraction protocols. As shown here, variation among replicates extracted with distinct DNA extraction protocols can be statistically significant. This is why comparing data from studies using different DNA extraction methods should be avoided (de la Cuesta-Zuluaga and Escobar 2016).

The other factor that more severely affected our results was the freezing and thawing of samples. This can lead to shifts in microbial community structure and abundance because bacteria are sensitive to long-term freezing and thawing (Pulido-Chavez et al. 2024). This is particularly relevant in studies where samples undergo repeated freeze–thaw cycles, which can alter the microbial profiles and affect the precision of microbiome analyses. For instance, in a postmortem study, thawing increased microbial diversity and altered community composition (Pechal et al. 2017). Similarly, frozen fecal samples yielded higher amounts of DNA from Gram-positive bacteria, probably because the freeze–thaw cycle disproportionately affects the Gram-positive cell wall (Bahl et al. 2012). This is likely the reason why repeated freeze–thaw cycles significantly affected the taxonomic and functional composition of microbiomes, with Gram-positive *Firmicutes* (*Bacillota*) and *Actinobacteria* (*Actinomycetota*) becoming more abundant (Poulsen et al. 2021). Repeated freeze–thaw cycles affect rare members of the microbiota, leading to significant distortions in microbial profiles after multiple cycles (Cuthbertson et al. 2015). Storage conditions also influence the impact of thawing. Samples stored at −80 °C seem more robust against thawing than samples stored at −20 °C (Pulido-Chavez et al. 2024). It is, therefore, recommended to minimize freeze–thaw cycles and ensure consistent storage conditions to maintain data integrity (Poulsen et al. 2021; Superdock et al. 2023).

Other factors evaluated in this study had smaller impacts on gut microbiota inference. Sample homogenization critically limits an experiment’s throughput due to the sample’s processing and the equipment’s sanitation. Some studies have shown that feces homogenization reduces intra-sample variability (Gorzelak et al. 2015; Jones et al. 2015) and increases replicability (Chiu et al. 2024). However, in our case, we did not find evidence that sample homogenization significantly affected gut microbiota profiles.

Variability introduced at library preparation and sequencing was also evaluated. We did not aim to evaluate different library preparation protocols or sequencing technologies. These are known to introduce biases that affect accuracy, precision, and comparability of metagenomic data (Tremblay et al. 2015; D’Amore et al. 2016; Sato et al. 2019; Winand et al. 2019; Tourlousse et al. 2021). A recent study comparing several protocols showed that library amplification by PCR, the one employed in the 16S rRNA methodology, led to higher variability than protocols evaluated in a PCR-free format. This was especially relevant for low DNA inputs and a high number of PCR cycles (Tourlousse et al. 2021). Evaluation of multiple library preparation kits for Illumina platforms highlighted the importance of selecting proper library preparation kits according to the purposes and targets of sequencing. This is because organisms with high GC content are more prone to errors introduced during library preparation (Jones et al. 2015; Bowers et al. 2015; Sato et al. 2019). In addition, different sequencing platforms had different error rates (Schloss et al. 2016; D’Amore et al. 2016; Winand et al. 2019). Our analysis aimed to assess variability with a single, standardized protocol for library preparation followed by Illumina MiSeq sequencing. It showed that repeatability at this level was very high.

After evaluating critical factors affecting the method’s repeatability, we evaluated our protocol concerning its variability within a single laboratory, that is, its intermediate precision. This level of technical precision has been rarely reported in large consortium studies but is essential for longitudinal and comparative research. We particularly focused on two factors that are more likely to vary within a single laboratory: the DNA extraction kit lot and the laboratory analyst handling samples. We found that the intermediate precision of our protocol was very high. Similar to our results, it was shown that microbiota measurements performed by changed reagent lots and laboratory analysts were only slightly higher than measurements between technical replicates (Tourlousse et al. 2021).

Previous studies have established precision benchmarks for microbiome research. A CV < 5% to 10% is generally used as a benchmark for intra-sample variability in most analytical methods. However, in the case of microbiome research, a target value of 30% for fecal samples was recently set (Tourlousse et al. 2021). For the intermediate precision, a CV < 10% to 15% is generally considered an acceptable benchmark, while Tourlousse et al. (2021) set a target value of 30% in microbiome studies with fecal samples. In our study, intra-sample variability was below 10% and inter-sample variability remained below 15% in nearly all cases, and CV values were consistently lower than 30%. These low variability metrics indicate a high degree of repeatability and robust intermediate precision of the method. Importantly, such low values suggest that technical variability contributes minimally to the overall variance of the data, thereby increasing confidence that observed differences between samples reflect true biological variation rather than methodological noise. This level of precision is particularly relevant for studies aiming to detect subtle changes in microbial community composition, to perform longitudinal or multicenter comparisons, or to evaluate the effects of interventions, where reproducibility and comparability across runs and operators are critical.

Considering analytical performance and processing time, we developed our own SOP for the 16S rRNA gene sequencing methodology for human gut microbiota characterization. The complete SOP can be found in Supplementary File 1. Briefly, it consists of self-collecting a fecal sample, refrigerating it, and transporting it to the laboratory within 4 h, similar to what is recommended by the International Human Microbiome Standards SOP 02 (Doré et al. 2015). In the laboratory, non-homogenized aliquots of fresh samples are employed for DNA extraction, using the adapted protocol of the Qiagen Blood & Tissue Kit for animal fecal tissues (Shanmuganandam 2019). Total DNA is quantified using fluorometry, diluted to 3 ng/μL, and used for library preparation and sequencing. Library preparation and sequencing followed an established SOP (https://github.com/SchlossLab/MiSeq_WetLab_SOP). Processing of the FASTQ files was performed following Mothur’s MiSeq SOP.

Our study makes a significant contribution to the microbiome field. We performed a rigorous analytical validation by establishing an SOP and specifically measuring its repeatability and intermediate precision. The presented SOP demonstrated high repeatability, high intermediate precision, and high sensitivity, achieving an LOQ as low as 11 rarefied reads, corresponding to a CV of 30%. By determining the method’s LOQ, we fill a common gap in the current microbiome literature, providing a concrete threshold for determining which low-abundance taxa can be reliably reported. The proposed SOP can be readily implemented in research laboratories that do not rely on sophisticated instrumentation. Beyond the core equipment typical of a molecular biology laboratory, it requires only a bead beater for sample homogenization, which can be acquired at a cost as low as USD 1,000. Moreover, all steps included in the SOP can be carried out by a trained laboratory technician. In our experience, this workflow enables the processing of approximately 100 samples per week by a single technician. Scaling up to accommodate larger sample numbers may require additional personnel or the incorporation of automation for sample handling.

The SOP mentioned above allowed quantifying variability in gut microbiota profiles among individuals and over time. Consistent with previous studies, we found notable inter-individual differences in gut microbiota diversity and composition (The Human Microbiome Project Consortium et al. 2012; Falony et al. 2016). Importantly, we found that there was general stability in the gut microbial community of the individual donors in the time window evaluated (one week). This is consistent with previous studies showing that human-associated microbial communities are generally stable, although they can be quickly and profoundly altered by changes in lifestyle and disease (David et al. 2014a, b; Jie et al. 2017).

In summary, our study contributes to the microbiome field by identifying specific technical drivers of variability of a specific 16S rRNA gene sequencing methodology for human gut microbiota characterization within a single, controlled laboratory to ensure the method’s reliability for routine use. It indicates that the DNA extraction protocol and sample thawing are the factors with the greatest impact on the repeatability and precision of 16S rRNA gene sequencing for human gut microbiota characterization. Assessing the method’s accuracy remains an important next step, although it is inherently challenging, as commonly used technical controls of known composition (e.g., cell mock communities) do not adequately capture the complex matrix effects that are highly relevant in fecal sample analysis. The SOP proposed here demonstrated excellent repeatability and strong intermediate precision, with CVs substantially lower than previously suggested target thresholds for fecal microbiome studies. We anticipate that this SOP can be readily adopted in most research laboratories and encourage its use and dissemination within the microbiome research community. More broadly, our findings highlight the critical importance of systematically quantifying variability introduced at each step of laboratory workflows to ensure methodological rigor. Such efforts are essential to improve the reproducibility, robustness, and generalizability of human microbiome research, ultimately leading to more reliable biological interpretations and stronger translational potential.

## Supplementary Information

Below is the link to the electronic supplementary material.

ESM 1 PDF (975 KB)

ESM 2 XLSX (730 KB)

ESM 3 XLSX (339 KB)

ESM 4 XLSX (68.9 KB)

ESM 5 XLSX (332 KB)

ESM 6 XLSX (341 KB)

ESM 7 XLSX (329 KB)

ESM 8 XLSX (392 KB)

ESM 9 XLSX (28.6 KB)

## Data Availability

Raw reads were deposited in the Short Read Archive (NCBI) under BioProject PRJNA1213012. The code to reproduce statistical analyses is available at https://github.com/jsescobar/attributes_16SrRNA .
