# Supplementary material for: Analytic attributes of the 16S rRNA gene sequencing methodology for human gut microbiota characterization
Source: Appl Microbiol Biotechnol. 2026 May 22;110:217. doi: 10.1007/s00253-026-13844-8 (PMC13375765; doi:10.1007/s00253-026-13844-8)
Supplement: Supplementary file 1 [file 253_2026_13844_MOESM1_ESM.pdf]

STANDARD OPERATING PROCEDURE

16S rRNA Gene Sequencing Methodology for Human Gut Microbiota Characterization

Objective: To outline the general guidelines for the collection and processing of fecal samples, fecal DNA extraction, and the shipment of DNA samples to an external sequencing laboratory.

Principle: Standardize all procedures related to the 16S rRNA gene sequencing methodology for human gut microbiota characterization in the Vidarium laboratory, with the aim of ensuring reliable, repeatable, and reproducible results.

1. Fecal Sample Collection

Materials and Equipment

Fecal Collection Kit:

- Sterile sample collection container (a).
- Fecal sample collection paper (Raku-Ryu Cup Wide) (b).
- Gloves (c).
- Wood tongue depressors (d).
- Bag for biological waste disposal (e).
- Disposable paper towel (f).
- Ziploc bag (not shown).

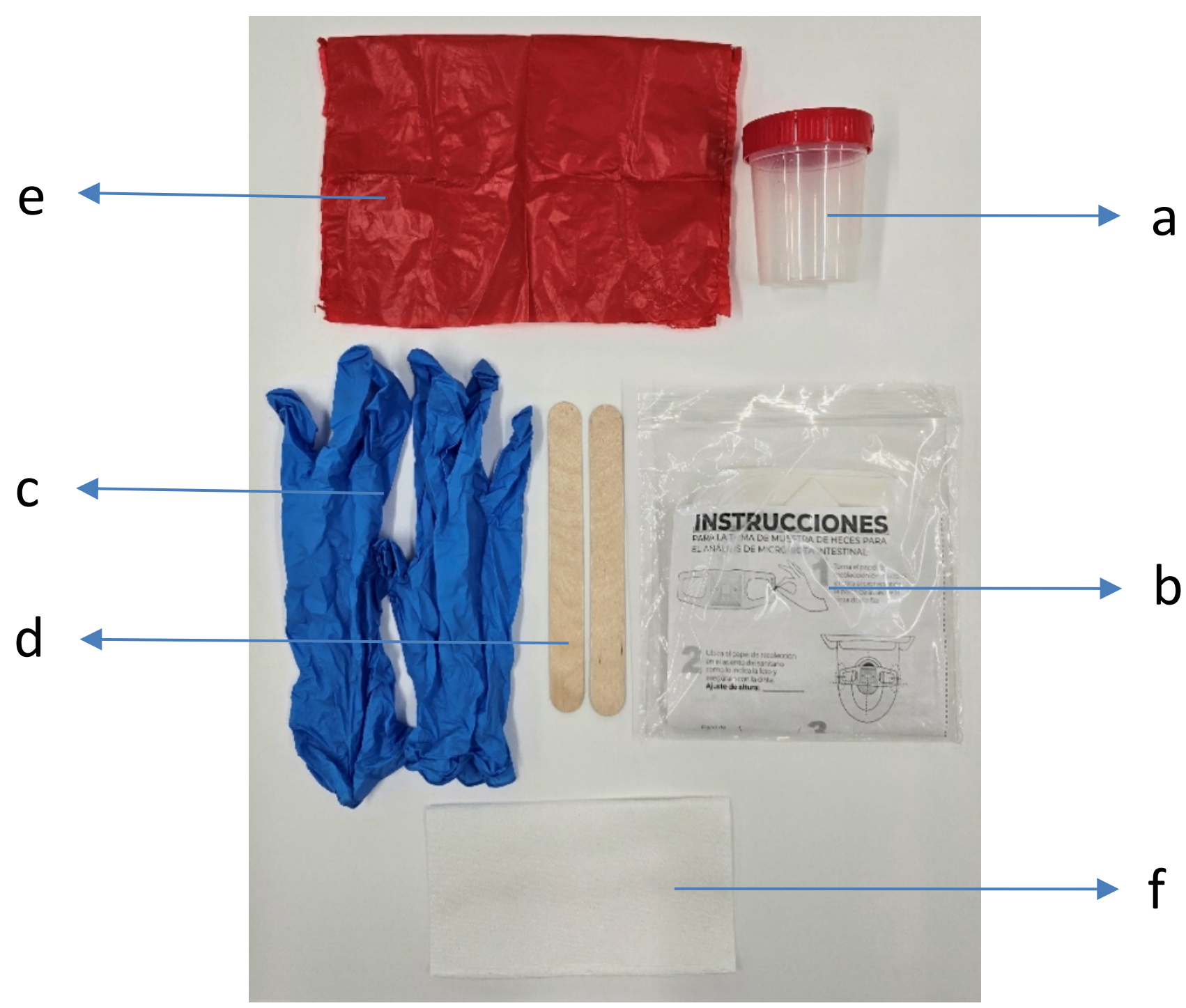

b

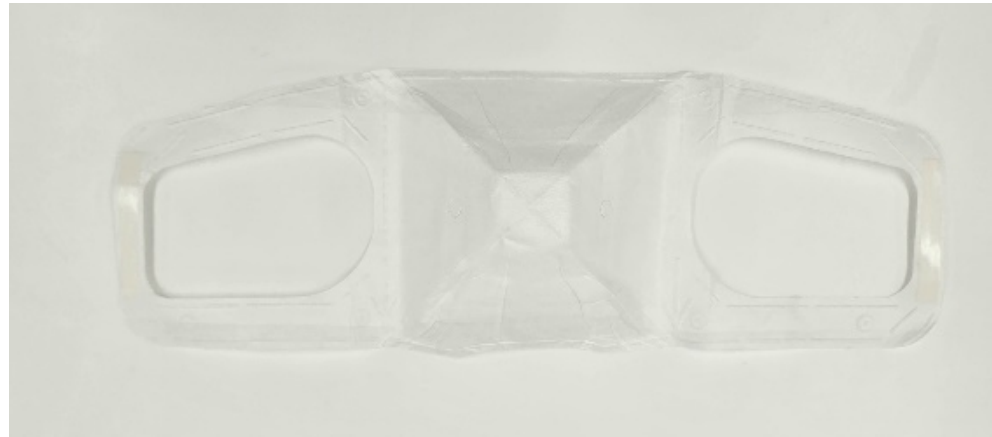

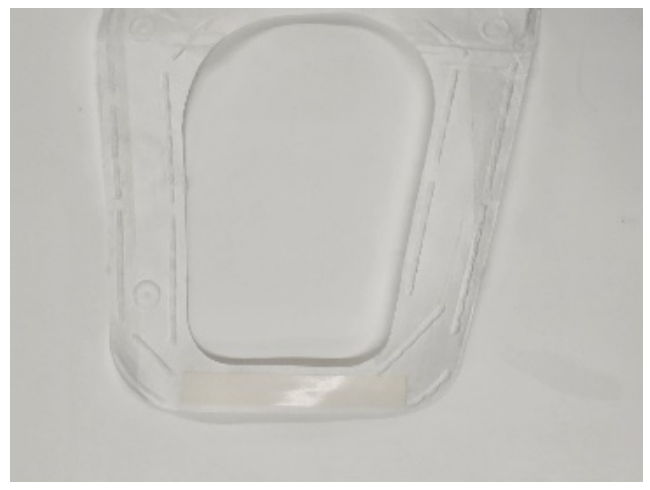

Cooler.
Refrigerant gel packs.

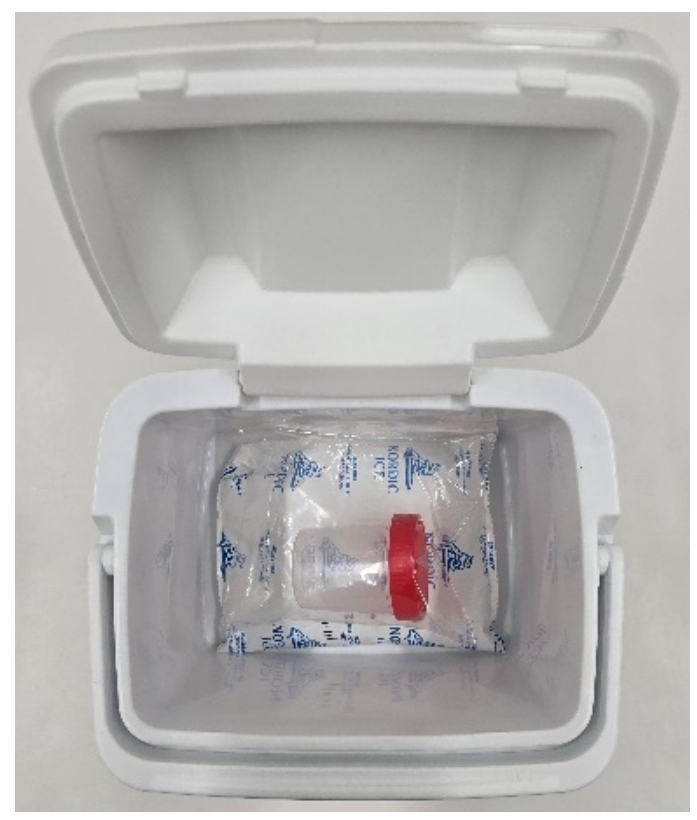

Instructions for fecal sample collection:

1. Make sure the refrigerant gel pack is completely frozen. If not, store it in the freezer long enough before collecting the sample.

1. Near the toilet, open the Ziploc bag containing the elements of the fecal collection kit and place them on a clean and dry surface that allows for easy handling.

2. Put on the gloves and unfold the stool sample collection sheet. Carefully remove the protective cover from the double-sided tape on the back of the device handles.

3. Lift the toilet seat lid. Make sure the toilet seat is clean and dry.

4. Place the fecal sample collection paper on the seat as shown in the photo. Secure it by attaching the tape from the handles around the seat, ensuring it holds the feces, does not shift, and does not fall due to its weight. The collection paper must not come into contact with the toilet water. Lower the toilet seat lid.

5. Once the stool is deposited on the collection paper, use the two tongue depressors to collect portions of the feces until filling 3/4 of the sterile collection container. Make sure that the stool sample does not come into contact with water, urine, or blood at any time during the collection process. Dispose of the tongue depressors in the provided red plastic bag.

6. Properly close the container, clean it with the provided paper towel, and dispose of the towel in the red bag. Place the sample container back inside the Ziploc bag. Lift the toilet lid and peel off the tape that attaches the collection paper to the toilet. Let it fall along with its contents into the toilet. Flush the water.

7. Place the container with the sample inside the cooler, along with the refrigerant gel pack. Properly close the cooler for transport to the laboratory. Remove your gloves and place them in the red bag, seal the bag, and dispose of it.

8. Wash your hands with water and soap. Contact the laboratory so that the staff can coordinate with you the sample delivery within a maximum of 4 hours.

2. Processing of Stool Samples in the Laboratory

Materials and Equipment

- Lab coat.
- Face mask.
- Safety goggles.
- Label printer.
- Laboratory fume hood.
- Gloves.
- Kraft paper.
- Paper towels.
- 70% alcohol.
- Sterile sample straws or 2 mL microtubes.
- Sterile wooden sticks.
- Parafilm.
- Sterile Falcon tubes.
- Red bag for waste disposal.
- Ultra-low temperature storage boxes.
- Ultra-low temperature freezer.

Instructions for aliquoting and storing stool samples in the laboratory

1. Verify that the cold chain has been maintained.

2. Assign an internal code to the sample and complete the intake form (internal form containing the sample code, date and time of receipt, information of the person receiving the sample, and the person who will aliquot it).

3. Generate the corresponding labels, and label the tubes and materials needed for aliquoting and storing the sample.

4. Put on gloves. Turn on the extraction cabinet and disinfect its surfaces with 70% alcohol. Cover the work area with Kraft paper.

5. Place all the materials for aliquoting inside the cabinet. Ensure that paper towels and a container with a red bag for waste disposal are available.

6. If you are going to use sterile straws, follow the process illustrated in the following image. Open the container holding the sample, take a straw, and press it firmly against the fecal matter so that the straw begins to fill with the stool. Press in different areas of the sample, ensuring that no air remains. Fill four straws and seal the edges with Parafilm. Clean the straws with a paper towel and insert them into sterile Falcon tubes. Store them in a cryobox at -80°C.

7. If using 2 mL microtubes, open the container holding the sample, take a sterile wooden stick, and homogenize it properly. Use the stick to transfer portions of the sample into five 2 mL tubes, ensuring they are completely filled. Organize the tubes in cryoboxes and store them at -80°C.

8. Properly discard the contaminated material, remove the Kraft paper, and disinfect the surfaces of the hood again with 70% alcohol. Remove your gloves and discard them.

3. Fecal DNA extraction

Materials and Equipment

- Bead beater.
- Label printer.
- Laboratory fume hood.
- Gloves.
- Disposable bouffant cap.
- Face mask.
- Safety goggles.
- Lab coat.
- Kraft paper.
- Paper towels.
- Aliquoted stool samples stored at -80 °C.
- Sterile wooden sticks.
- Sterile scissors.
- Ice.
- Analytical balance.
- DNeasy Blood and Tissue Kit.
- RNase A.
- Sterile microtubes for bead beating, pre-filled with 1 g of 0.1 mm and 0.5 mm high-impact zirconium beads.

DNA Extraction from Fecal Samples Using the DNeasy Blood & Tissue Kit (Qiagen)

1. Turn on the extraction cabinet and disinfect its surfaces with 70% alcohol. Cover the work area with Kraft paper. Place all the materials required inside the cabinet. Ensure that paper towels and a container with a red bag for waste disposal are available.

2. If samples were aliquoted in a straw and frozen, remove the straw quickly from -80 °C. Inside the fume hood, cut a portion of the straw and place it into a sterile 2 mL microtube. Reseal the remaining straw with Parafilm and return it to -80 °C. From the cut portion, release the sample and weigh 150–200 mg. Always keep the samples on ice. Record the exact weight in the corresponding form. Change gloves between samples to avoid cross-contamination.

3. If samples were aliquoted in 2 mL microtubes, place them on ice until they reach room temperature.

4. Weigh 150-200 mg of fecal tissue and transfer it into a sterile 2 mL tube containing zirconium beads (0.1 + 0.5 mm, TriplePure M-Bio Grade, Benchmark). Record the exact weight in the corresponding form.

In the DNA extraction area:

5. Add 200 µL of ATL buffer and 20 µL of Proteinase K.

6. Homogenize the mixture twice for 45 s at maximum speed using a bead disruptor (BeadBlaster 24, Benchmark, China).

7. Incubate the sample at 56°C for 10 min.

8. Add 4 µL of RNase A (100 mg/mL) (Qiagen), vortex briefly, and incubate at room temperature for 5 min.

9. Centrifuge at 13,300 rpm for 7 min.

10. Carefully transfer the supernatant to a new 1.5 mL tube, avoiding pellet disturbance.

Purification using DNeasy MiniSpin Columns

11. Add 200 µL of AL buffer and mix by vortexing.

12. Add 200 µL of ethanol (96–100%) and vortex again.

13. Transfer the mixture to a DNeasy MiniSpin column placed in a collection tube.

14. Centrifuge at 13,300 rpm for 3 min. Discard the filtrate and collection tube.

15. Place the column into a new collection tube and add 500 µL of AW1 buffer.

16. Centrifuge at 10,000 rpm for 1 min. Discard the filtrate and collection tube.

17. Add 500 µL of AW2 buffer to the column.

18. Centrifuge at 13,300 rpm for 3 min to dry the membrane. Discard the filtrate and collection tube.

19. Transfer the column to a new 1.5 mL tube and centrifuge at 13,300 rpm for 1 min.

20. Discard the tube and place the column into a new 1.5 mL tube.

21. Pipette 25 µL of AE buffer onto the membrane, incubate at room temperature for 1 min, and centrifuge at 10,000 rpm for 1 min to elute the DNA.

22. Repeat step 19 with an additional 25 µL of AE buffer to obtain a final volume of 50 µL.

23. Label the tubes accordingly. Proceed with DNA quantification by fluorometry. Store the extracted DNA at -20°C until further use.

DNA Extraction from Fecal Samples Using the Modified Protocol to Maximize Gram-Positive DNA Recovery with the DNeasy Blood & Tissue Kit (Qiagen)

a. After step 2 of the "DNA Extraction from Fecal Samples Using the DNeasy Blood & Tissue Kit (Qiagen)" protocol, add 200 µL of lysis buffer (20 mM Tris-Cl, pH 8.0, 2 mM sodium EDTA, 1.2% Triton X-100, lysozyme to 20 mg/mL).

b. Incubate at 37°C for 30 min.

c. Proceed with step 3 of the standard protocol (addition of ATL buffer and Proteinase K) and follow the remaining steps as previously described.

4. DNA Quantification by Fluorometry

Materials and Equipment

- Gloves.
- Lab coat.
- Disposable bouffant cap.
- Face mask.
- DNA samples.
- Ice.
- Sterile nuclease-free water
- 0.5 mL thin wall PCR tubes with flat cap, clear.
- Quantus™ Fluorometer (Promega).
- QuantiFluor® ONE dsDNA System.

Procedure

In the DNA quantification area:

1. Keep DNA samples on ice.

2. Follow the manufacturer's instructions, starting with equipment calibration using the DNA standard.

3. Perform concentration measurements using 2 µL of DNA per sample, and adjust the instrument settings as needed.

4. Record the results in the corresponding form.

5. If necessary, dilute the DNA to the desired concentration (usually 3 ng/µL).

6. Check the concentration of selected dilutions. Record the results.

7. Store the extracted DNA and dilutions at -20°C until further use.

5. **Plate preparation for sequencing submission**

Materials and Equipment

- Gloves.
- Lab coat.
- Disposable bouffant cap.
- Face mask.
- DNA sample dilutions
- Ice.
- Sterile 96-well plate.
- Sterile Snap-on lids for 96-well plates.
- Plate map indicating the location of each sample.
- Parafilm.
- Two pieces of cardboard the size of the plate.
- Tape.
- Ziploc bag.

Procedure

1. Work in a clean, designated, and disinfected area. Thaw the samples to be loaded on ice. Arrange them in a rack according to the pre-designed loading map. Before loading the plate, have a second person verify that the sample order is correct.

2. Load the plate by columns, starting from column 1 to column 12. After completing each column, immediately cover the wells.

3. Take each microtube containing a sample and vortex it for 5 seconds. Pipette the required volume (usually 20 µL) and dispense it into the corresponding well according to the loading map. A second person must verify that the sample is being placed in the correct well.

4. Once the plate has been loaded, seal the edges of the lid to the plate using Parafilm. Label the plate.

5. Wrap the plate in a paper towel to absorb any external moisture. Then, place it between two pieces of cardboard and secure them to the plate with tape (in a sandwich-like arrangement). This is to protect the integrity of the plate during transport. Label the plate again.

6. Place the plate inside a Ziploc bag and store it at -80 °C until shipment. Transport the plate to the sequencing laboratory on dry ice.

References

- *DNeasy® Blood & Tissue Handbook*. Qiagen. https://www.qiagen.com
- *QuantiFluor® ONE dsDNA System: Instructions for use of products E4871 and E4870* [Technical manual]. Promega. https://www.promega.com
- Romano, K.A., Dill-McFarland, K.A., Kasahara, K. *et al.* Fecal Aliquot Straw Technique (FAST) allows for easy and reproducible subsampling: assessing interpersonal variation in trimethylamine-*N*-oxide (TMAO) accumulation. *Microbiome* 6, 91 (2018). https://doi.org/10.1186/s40168-018-0458-8.
- University of Michigan Microbiome Core. (2021). *Protocol for shipping plates to the Microbiome Core* (Version: 2021). UMich Microbiome Core Protocols. https://microbiome.med.umich.edu
